# Hydrophobic ionic liquids for quantitative bacterial cell lysis with subsequent DNA quantification

**DOI:** 10.1007/s00216-016-0112-x

**Published:** 2016-12-21

**Authors:** Sabine Fuchs-Telka, Susanne Fister, Patrick-Julian Mester, Martin Wagner, Peter Rossmanith

**Affiliations:** 1 Christian Doppler Laboratory for Monitoring of Microbial Contaminants, University of Veterinary Medicine, Veterinärplatz 1, 1210 Vienna, Austria; 2 Institute for Milk Hygiene, Milk Technology, and Food Science, University of Veterinary Medicine, Veterinärplatz 1, 1210 Vienna, Austria

**Keywords:** Ionic liquids, DNA extraction, Quantitative PCR, *Salmonella* Typhimurium, *Escherichia coli*, Solvent extraction, Two-phase system

## Abstract

DNA is one of the most frequently analyzed molecules in the life sciences. In this article we describe a simple and fast protocol for quantitative DNA isolation from bacteria based on hydrophobic ionic liquid supported cell lysis at elevated temperatures (120–150 °C) for subsequent PCR-based analysis. From a set of five hydrophobic ionic liquids, 1-butyl-1-methylpyrrolidinium bis(trifluoromethylsulfonyl)imide was identified as the most suitable for quantitative cell lysis and DNA extraction because of limited quantitative PCR inhibition by the aqueous eluate as well as no detectable DNA uptake. The newly developed method was able to efficiently lyse Gram-negative bacterial cells, whereas Gram-positive cells were protected by their thick cell wall. The performance of the final protocol resulted in quantitative DNA extraction efficiencies for Gram-negative bacteria similar to those obtained with a commercial kit, whereas the number of handling steps, and especially the time required, was dramatically reduced.

**Electronic supplementary material:**

The online version of this article (doi:10.1007/s00216-016-0112-x) contains supplementary material, which is available to authorized users.

**Graphical Abstract:**
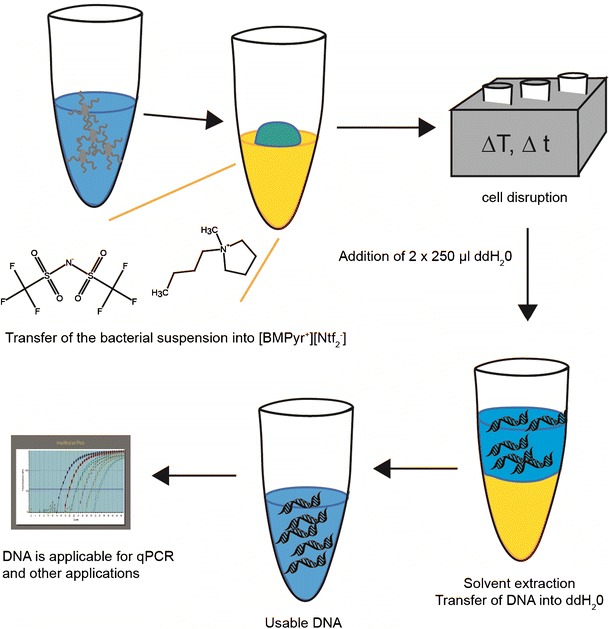
After careful evaluation of five hydrophobic ionic liquids, 1-butyl-1-methylpyrrolidinium bis(trifluoromethylsulfonyl)imide (*[BMPyr* ^*+*^ *][Ntf*_*2*_^*-*^ *]*) was identified as the most suitable ionic liquid for quantitative cell lysis and DNA extraction. When used for Gram-negative bacteria, the protocol presented is simple and very fast and achieves DNA extraction efficiencies similar to those obtained with a commercial kit. *ddH*_*2*_ *O* double-distilled water, *qPCR* quantitative PCR

## Introduction

In the life sciences, particularly in molecular biology, one of the most important classes of molecules are nucleic acids, especially DNA. Because of their prominent role in all known living organisms, various methods have been developed to analyze them to address biological and biochemical questions in clinical diagnostics, food safety, genomics, microbiology, and environmental science [1–7]. Today bioanalytical techniques, including PCR, quantitative PCR (qPCR), cloning, and sequencing methods are capable of analyzing very small amounts of target molecules (in the case of qPCR even down to one target molecule), which as a consequence must be of high purity and quality [8, 9]. State-of-the-art DNA extraction methods either are mostly column based or use functionalized magnetic particles, which renders them laborious to perform and difficult to automate [10].

Novel approaches utilizing ionic liquids (ILs) have also been successfully applied to DNA extraction, further improving the previously established systems, via a second hydrophobic extraction phase, solid-phase microextraction, or magnetic IL extraction [11–14].

What all these extraction methods have in common is that they can be separated into two general steps: the first step is lysis of the cell; the second step is separation and purification whereby the desired nucleic acid is extracted from the aqueous cell debris and sample residues. It is of the utmost importance to differentiate between these two general steps in DNA extraction as they are often incorrectly conflated; most commercial and recently developed methods focus only on the extraction step while ignoring cell lysis. In respect of the various application fields, it has to be made clear that crude DNA extracts are suitable for only very few applications, and in most cases quantitative nucleic acid isolation is necessary [1, 10]. This is especially true in diagnostics, where legal regulations define the number of pathogens permitted to be present in the sample or only a small number of target cells are present within a huge background [1]. This problem is very well reflected by the actual time required for the two separate steps in commercial DNA purification kits for bacteria, such as the NucleoSpin® tissue kit, which was used in this study. According to the manufacture’s specifications, the cell lysis step with the NucleoSpin® tissue kit for bacteria takes between 2.5 and 20 h, whereas the actual silica-column-based DNA extraction and purification takes only approximately 5 min per sample. In this context, cell lysis is the most crucial step, and the time required for its execution is crucial. Because of the characteristics of ILs, such as their ability to dissolve biomass, their high thermal stability, and their ability to preserve DNA integrity in the presence of nucleases, they have recently been recognized to improve DNA extraction steps, cell lysis, and purification. To the best of our knowledge, hydrophilic ILs have been most successful for cell lysis [15, 16], whereas hydrophobic ILs have proven useful for extraction of DNA from crude cell extracts [11, 17]. The downside of hydrophilic ILs is their possible interference or even complete inhibition of subsequent molecular-biological methods, such as qPCR [18], and therefore must be removed before analysis, and quantitative back-extraction of DNA from hydrophobic ILs is also challenging.

We report here a new method, based on hydrophobic ILs, for fast and quantitative bacterial cell lysis with subsequent DNA extraction followed by downstream molecular-biological techniques such as qPCR. A set of five hydrophobic ILs were carefully evaluated for their potential as cell lysis/DNA extraction liquids, and the whole method was further tested and improved for the final candidate. The final method permits quantitative DNA extraction from Gram-negative bacteria with performance equivalent to that of a commercial kit in only a fraction of the time.

## Materials and methods

### ILs, test kits, and chemicals

ILs *N*-butyl diethanol ammonium bis(trifluoromethylsulfonyl)imide ([DEBA^+^][Ntf_2_^-^]), trihexyltetradecylphosphonium tris(pentafluoroethyl)trifluorophosphate ([N_6,6,6,14_^+^][FAP^-^]), trihexyltetradecylphosphonium bis(trifluoromethylsulfonyl)imide ([N_6,6,6,14_^+^][Ntf_2_^-^]), 1-butyl-1-methylpyrrolidinium bis(trifluoromethylsulfonyl)imide ([BMPyr^+^][Ntf_2_^-^]), and 1-hexyl-3-methylimidazolium tris(pentafluoroethyl)trifluorophosphate ([C_6_mim^+^][FAP^-^]), and precoated cellulose thin-layer chromatography (TLC) plates (20 × 20) were provided by Merck (Darmstadt, Germany). The NucleoSpin® tissue kit was provided by Macherey-Nagel (Düren, Germany). Diethylpyrocarbonate-treated water, primer, and probes were provided by Invitrogen (Lofer, Austria). MgCl_2_ (purity 98% or greater, CAS 7786-30-3), KCl (purity 99% or greater, CAS 7447-40-7), NaCl (purity 98% or greater, CAS 7647-14-5), amino acids as their l enantiomers (alanine, cysteine, glutamine, histidine, leucine, phenylalanine, tyrosine, and tryptophan, purity 99% or greater), monosaccharides as their d enantiomers (glucose, fructose, mannose, and galactose), and Taq polymerase were provided by Fisher Scientific (Vienna, Austria).

### Bacterial strains and culture conditions

*Salmonella* Typhimurium (NCTC 12023) and *Escherichia coli* TOP10F were used as model organisms for Gram-negative bacteria. *Listeria monocytogenes* EGDe (1/2a, internal number 2964) was used as a model organism for Gram-positive bacteria. All strains were maintained at -80 °C with use of MicroBank technology (Pro-Lab Diagnostics, Richmond Hill, Canada). *L. monocytogene*s, *Salmonella* Typhimurium, and *E. coli* TOP10F are part of the collection of bacterial strains at the Institute of Milk Hygiene, Department of Veterinary Public Health and Food Science, University of Veterinary Medicine (Vienna, Austria). All bacterial strains were grown overnight in tryptone soy broth with 0.6% (w/v) yeast extract (Oxoid, Basingstoke, UK) at the optimal growth temperature of 37 °C.

### Quantitative PCR setup and DNA standard preparation

An Mx3000 qPCR thermocycler (Stratagene, La Jolla, CA, USA) was used for the qPCR experiments; the 25-μl reaction mixture contained 5 μl DNA template. The qPCR results were expressed as bacterial cell equivalents, and all qPCR assays were performed in duplicate. Quantitative PCRs (qPCRs) for quantification of *L. monocytogenes* [19], *Salmonella* Typhimurium [20], and *E. coli* [21] were performed as previously reported, and detailed information about the respective qPCR assays is summarized in the electronic supplementary material.

One milliliter of an overnight culture of each bacterial species was used for DNA isolation with the NucleoSpin® tissue kit, according to the manufacturer’s specifications. Determination of DNA concentration was performed by fluorimetric measurement with an Hoefer DyNA Quant 200 apparatus (Pharmacia Biotech, San Francisco, CA, USA). Dilution series (two to five dilutions) in 1× PCR buffer were made. These dilutions were quantified by qPCR, and the series were treated as a standard curve. The efficiency and the *R* ^2^ correlation statistic were calculated.

### Testing of inhibition of qPCR by ILs and evaluation of DNA distribution between aqueous and IL phases

We added 250 μL of double-distilled water (ddH_2_O) to 250 μl of the respective ILs. After agitation (30 s) and centrifugation (5 min, 2500 *g*) to obtain phase separation, the upper aqueous phase was transferred into a new tube. To measure the influence of solubilized or dispersed hydrophobic ILs in the aqueous phase, 5 μl of the aqueous phases was added to the master mix of the *Salmonella* Typhimurium qPCR already including 1 × 10^5^ *Salmonella* Typhimurium DNA copies.

For evaluation of DNA distribution, 250 μL of a 5 × 10^-3^ ng/μl solution of *Salmonella* Typhimurium DNA solution was added to 250 μl of the respective ILs. After agitation (30 s) and centrifugation (5 min, 2500 *g*) to obtain phase separation, the upper aqueous phase was transferred into a new tube. The IL phase was again extracted with 250 μl ddH_2_O. The DNA concentration in both aqueous phases was measured by qPCR.

### Development of an apparatus for cell disruption and experimental setup

As small-volume containers (100 μl or less) for ILs, 0.3-ml glass microcartridges for screw thread bottles (40 mm × 6 mm; Fisherbrand, Fisher Scientific Austria, Vienna, Austria), equipment normally used for high-performance liquid chromatography measurements, were used. For heating, an aluminum block with drilled holes, fitting the dimensions of the microcartridges, was custom-manufactured and placed on a heating plate (IKAMAG® RCT; IKA-Labortechnik, Staufen im Breisgau, Germany) (see Fig. S1). The temperature was measured with a metal thermometer (Ama-digit ad 14 th, Amarell, Kreuzwertheim, Germany) directly in one reference vial containing IL placed in the aluminum block.

For the cell disruption experiments, overnight cultures of *Salmonella* Typhimurium or *E. coli* were harvested by centrifugation (5 min, 6000 *g*), washed three times with ddH_2_O, and resuspended in 1/20 of the initial volume in ddH_2_O. Ten microliters of the resuspended culture was placed into 100 μl of [BMPyr^+^][Ntf_2_^-^], covered with porous aluminum foil, and incubated for various times (1, 2, 5, and 30 min) and at different temperatures (80, 120, 150, and 180 °C). Following incubation, 250 μl ddH_2_O was added, and the two phases were mixed with a pipette and transferred into a 2-ml tube (Eppendorf, Hamburg, Germany). An additional 250-μl ddH_2_O aliquot was used as the vehicle to transfer the remnant into the tube. The tube contents were briefly vortexed, and the upper aqueous phase was used directly for qPCR measurements following rapid, spontaneous IL separation despite not introducing an additional centrifugation step.

Ten microliters of the same bacterial suspension used in the cell disruption experiments was used for DNA isolation with the NucleoSpin® tissue kit in accordance with the manufacturer’s specifications. The final step of the protocol was modified. Instead of one wash with 100 μl prewarmed (70 °C) elution buffer BE, two washes with 50 μl prewarmed ddH_2_O were used for DNA elution from the column [22]. Finally, 400 μl of ddH_2_O was added to achieve the same volume as in the cell disruption experiments. The samples were used directly for qPCR.

### Extraction of salts, sugars, and lipids

Aqueous solutions of the chloride salts of magnesium (30 ppm), potassium (300 ppm), and sodium (40 ppm) were prepared and vortexed in a ratio of 1:1 with [BMPyr^+^][Ntf_2_^-^]. These solutions, including controls, were diluted 100-fold and analyzed with a PerkinElmer 3030B atomic absorption spectrometer (PerkinElmer, Wellesley, MA, USA), according to the manufacturer’s instructions. Monosaccharide (d-glucose, d-fructose, d-mannose, d-galactose) solutions were prepared according to the protocol of Matissek et al. [23]. Solutions were either applied directly onto the TLC plate, vortexed in a ratio of 1:1 with [BMPyr^+^][Ntf_2_^-^] before application, or applied after an additional centrifugation step (5 min, 6000 *g*). TLC plates were developed according to the protocol for the identification of sugars by Matissek et al. [23]. Amino acid solutions (l-alanine, l-cysteine, l-glutamine, l-histidine, l-leucine, l-phenylalanine, l-tyrosine, and l-tryptophan) were prepared according to the method of Matissek et al. [23]. Either amino acid solutions were applied directly or a mixture of all eight amino acids was vortexed in a ratio of 1:1 with [BMPyr^+^][Ntf_2_^-^] with and without additional centrifugation (5 min, 6000 *g*) or preincubated (2 min, 140 °C) in a ratio of 1:1 with [BMPyr^+^][Ntf_2_^-^] with and without additional centrifugation for (5 min, 6,000 *g*). The TLC plates were developed according to the protocol for the identification of amino acids by Matissek et al. [23].

### Transmission electron microscopy

The protocol of Glauert et al. [24] was followed to prepare negative-stained ultrathin section samples. In brief, prefixation was achieved with 5% glutaraldehyde, and fixation was achieved with 1% osmium tetroxide. Samples were mixed with agar, dehydrated, and embedded. After polymerization, ultrathin sections with a thickness of 70–90 nm were cut, and staining of the samples for transmission electron microscopy was performed with phosphotungstic acid [24]. Negative-stained ultrathin section samples *of Salmonella* Typhimurium and *E. coli* were analyzed with a Zeiss (Vienna, Austria) EM900 transmission electron microscope after the respective cell disruption procedures had been conducted.

## Results and discussion

The development of an efficient cell lysis method for quantitative pathogen detection by the five hydrophobic ILs required evaluation of their DNA uptake capacity and their possible capacity for inhibition of downstream methods. In contrast to previously studied IL-based DNA extraction methods, the DNA intake capacity of the ILs should preferably be very small to minimize the need for multiple elution steps following cell lysis, and thereby reduce the need for complex handling and shorten the time required. Hydrophobic ILs often consist of cations possessing long alkyl side chains and/or of fluorinated anions, both which have been previously reported to interfere or inhibit enzymatic reactions such as PCR [17, 18]. Although several possible intervention strategies have recently been reported to protect PCR-based methods from inhibition by hydrophobic ILs [15, 17], limited solubility of ILs in the aqueous elution phase would be preferable.

### Quantitative PCR inhibition by hydrophobic ILs in the aqueous eluate

**Fig. 1:**
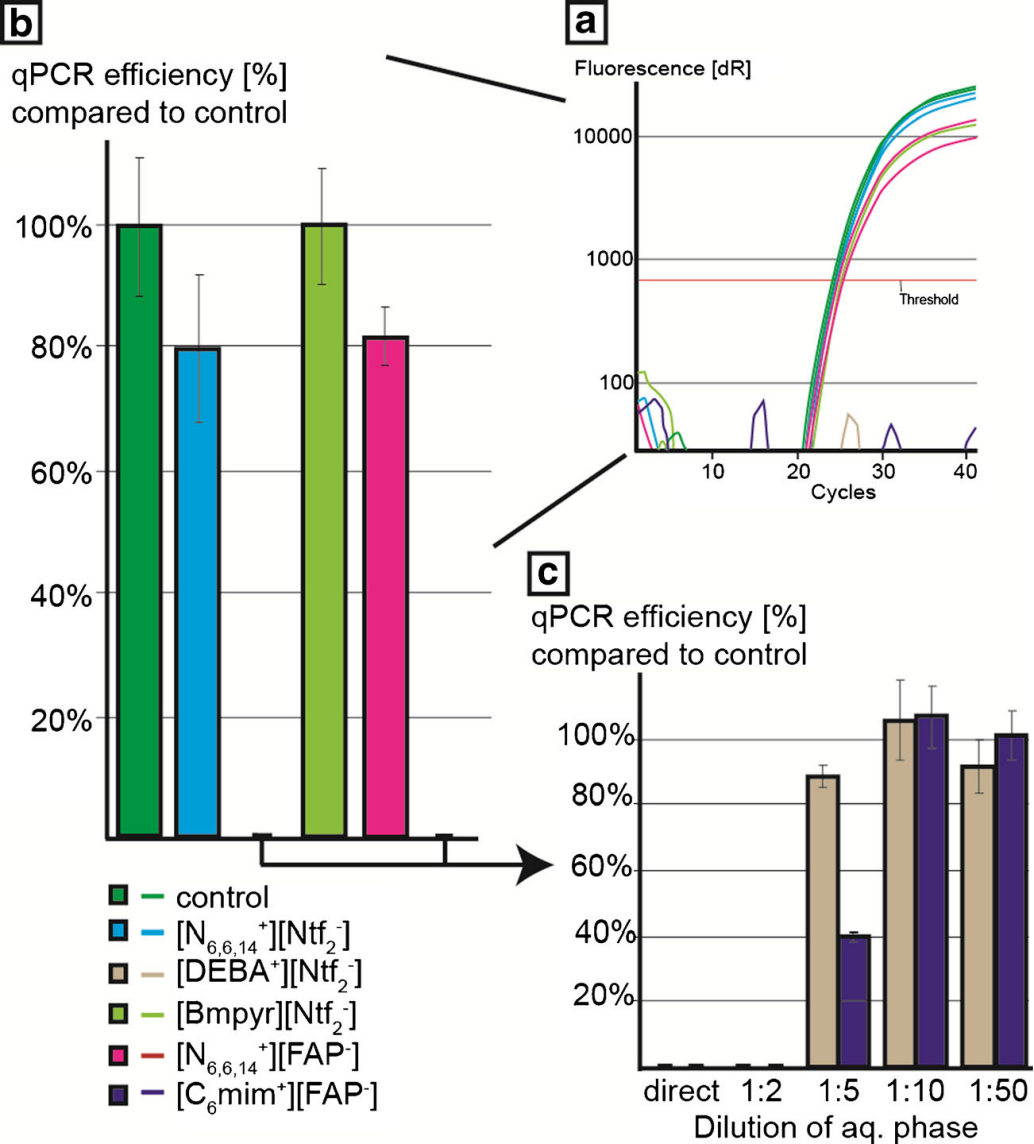
Quantitative PCR (*qPCR*) inhibition of aqueous phases caused by leaching of hydrophobic ionic liquids compared wit a control (*A*) and mean values with the respective standard deviation of three independent experiments (*B*). Dilutions of the aqueous phases extracted from trihexyltetradecylphosphonium tris(pentafluoroethyl)trifluorophosphate (*[N*_*6,6,6,14*_^*+*^ *][FAP* ^*-*^ *]*) and trihexyltetradecylphosphonium bis(trifluoromethylsulfonyl)imide (*[N*_*6,6,6,14*_^*+*^ *][Ntf*_*2*_^*-*^ *]*) in double-distilled water (ddH_2_O) and their respective mean inhibition values with the respective standard deviation of the qPCR (*C*). *[Bmpyr* ^*+*^ *][Ntf*_*2*_^*-*^ *]* 1-butyl-1-methylpyrrolidinium bis(trifluoromethylsulfonyl)imide, *[C*_*6*_ *mim* ^*+*^ *][FAP* ^*-*^ *]* 1-hexyl-3-methylimidazolium tris(pentafluoroethyl)trifluorophosphate, *[DEBA* ^*+*^ *][Ntf*_*2*_^*-*^ *] N*-butyl diethanol ammonium bis(trifluoromethylsulfonyl)imide

To investigate the applicability of the five hydrophobic ILs used in this study, IL-mitigated inhibition was tested directly with use of qPCR. To simulate extraction, the respective ILs were agitated for 30 s together with an equal volume of ddH_2_O, and after separation via centrifugation (5 min at 2500 *g*), the aqueous phase was tested for possible interference or inhibition of a *Salmonella* Typhimurium qPCR. In the case of [DEBA^+^][Ntf_2_^-^] and [C_6_mim^+^][FAP^-^], complete inhibition of the qPCR was observed when the aqueous phase, separated after extraction, was applied directly (Fig. 1, plots A and B). To prevent inhibition by these two ILs, it was necessary to make a tenfold dilution of the aqueous phase (Fig. 1, plot C). The respective aqueous phases for [N_6,6,6,14_^+^][FAP^-^] and [N_6,6,6,14_^+^][Ntf_2_^-^] resulted in a reduced qPCR efficiency of approximately 80%, indicating a slight inhibition of the enzymatic reaction due to the presence of IL. The only hydrophobic IL that did not cause any qPCR inhibition was [BMPyr^+^][Ntf_2_^-^], and therefore this IL could be used without additional purification or intervention methods.

### DNA uptake by hydrophobic ILs

**Fig. 2:**
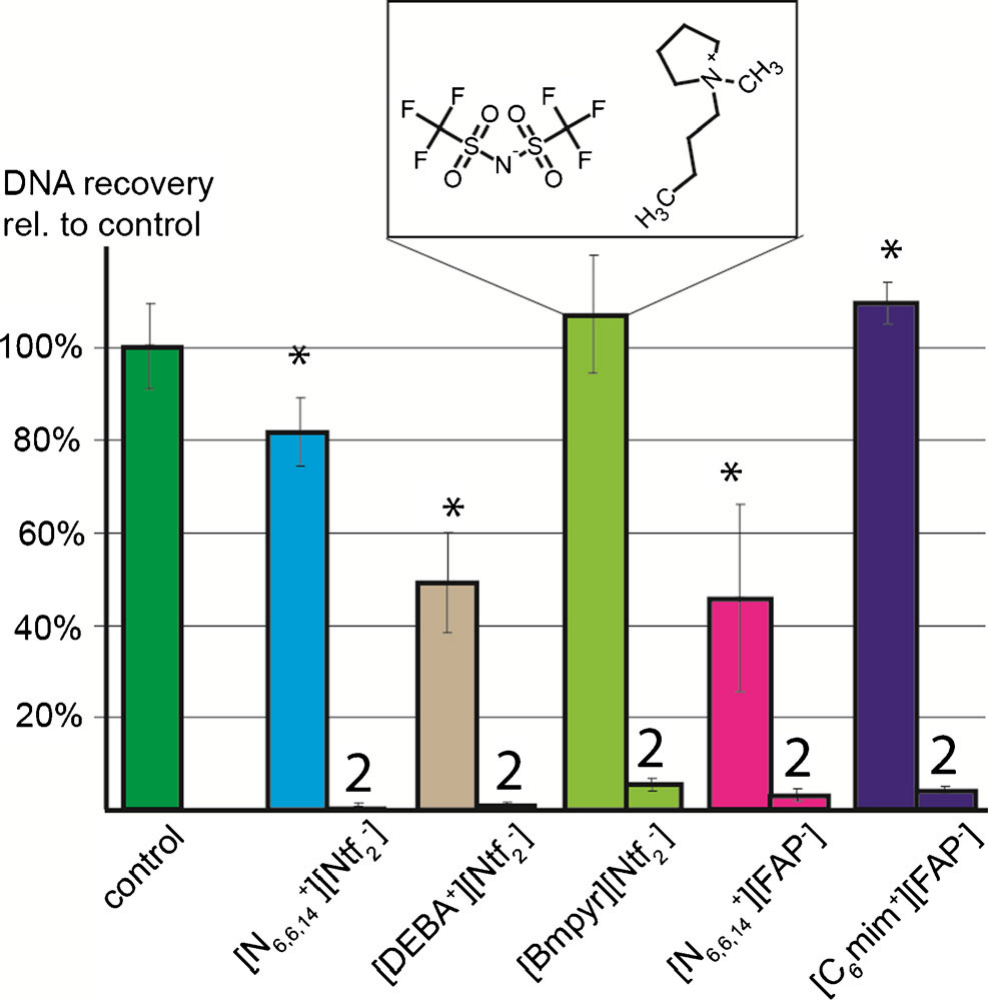
DNA quantity in the aqueous phase after extraction with ionic liquids compared with the initial input. Mean values with the respective standard deviation of three independent experiments are shown. The number *2* indicates the second extraction cycle. Samples that had to be diluted tenfold in ddH_2_O to avoid qPCR inhibition are indicated by an *asterisk. rel.* relative

As for the inhibition studies, genomic DNA of the Gram-negative bacterium *Salmonella* Typhimurium was selected as a model to measure the IL DNA uptake capacity. DNA in aqueous solution (5 × 10^-3^ ng/μl) was agitated for 30 s together with an equal volume of a particular IL. The respective DNA concentration in the aqueous phase directly after phase separation and after a second extraction step was determined by qPCR, and the results are shown in Fig. 2. Because of the inhibitory effects of IL remnants as determined in the previous experiments, respective aqueous solutions of [N_6,6,6,14_^+^][FAP^-^], [N_6,6,6,14_^+^][Ntf_2_^-^], [C_6_mim^+^][FAP^-^], and [DEBA^+^][Ntf_2_^-^] were diluted tenfold in ddH_2_O. For [N_6,6,6,14_^+^][FAP^-^] and [DEBA^+^][Ntf_2_^-^], an almost stoichiometric distribution of the genomic DNA between the IL phase and the aqueous phase was determined, which is in good accordance with the findings of previous studies using hydrophobic ILs to extract DNA from aqueous solution [11, 13, 17]. However, although desirable in such applications, DNA uptake is disadvantageous during lysis of bacterial cells as quantitative release of DNA from the IL phase is not possible, even after an additional extraction step. For [N_6,6,6,14_^+^][Ntf_2_^-^] and [C_6_mim^+^][FAP^-^], almost no DNA uptake into the IL phase was measured. However, as discussed, following extraction, for both ILs the aqueous phase had to be diluted to prevent qPCR inhibition secondary to IL in the aqueous eluate. No DNA uptake into the IL phase was observed for [BMPyr^+^][Ntf_2_^-^], and in contrast to the other ILs tested, the aqueous phase following extraction could be used directly without additional dilution.

### Uptake of cellular components by [BMPyr^+^][Ntf_2_^-^]

Although the main focus of this study was to investigate hydrophobic ILs for bacterial cell lysis and DNA extraction, secondary goals included the possibility of DNA purification from cellular debris and the extent of downstream inhibition. Hydrophobic ILs have been previously reported to take up selectively and thus purify DNA from crude cellular extracts [13, 19]. As previously indicated, for the proposed application of cell lysis and DNA extraction, selective DNA uptake would be disadvantageous. Nevertheless, although [BMPyr^+^][Ntf_2_^-^] was specifically selected because of its negligible DNA uptake, possible uptake of cellular components such as salts, lipids, carbohydrates, or proteins (all of which can inhibit downstream molecular-biological methods) was also analogously investigated. The presence of salts (i.e., KCl, NaCl, MgCl_2_) was analyzed by electrothermal atomic absorption spectroscopy (ET-AAS). The concentrations to be measured were chosen to be at least 100-fold above the limit of quantification of the ET-AAS device (300 ± 3 ppb for Mg; 2986 ± 5 ppb for K; 383 ± 6 ppb for Na). The results demonstrated no removal from the aqueous phase after phase extraction with [BMPyr^+^][Ntf_2_^-^] (Mg, 294 ± 3 ppb; K, 2972 ± 13 ppb; Na, 381 ± 6 ppb). For carbohydrates, lipids, and proteins, TLC methods were used for semiquantitative evaluation. Likewise, in the case of monosaccharides (glucose, fructose, galactose, and mannose), amino acids (see Figs. S3, S4), and lipids (results not shown), there was no uptake by [BMPyr^+^][Ntf_2_^-^] of any of the substances tested.

### Thermal bacterial cell lysis and DNA extraction by [BMPyr^+^][Ntf_2_^-^]

For actual disruption of cells, several approaches are commonly used, and can be classified as physical (e.g., boiling, grinding, osmotic shock, dry–thaw), chemical (e.g., chaotropic or chelating agents, detergent lysis, acid/base), or biological (e.g., enzymes such as lysozyme or proteinase K, phages) methods. Each of these methods has certain advantages and disadvantages [25], but for automation and to reduce the number of handling steps, either boiling or chemical lysis or a combination of both is most advantageous. Although boiling in water has been reported to be efficient for the extraction of nucleic acids from eukaryotic cell cultures, this technique does not lead to quantitative release from more complex matrices or especially bacterial cells. One of the main advantages of ILs in this regard is, of course, their excellent thermal stability and negligible vapor pressures, which permits the use of higher temperatures than would be possible with aqueous solutions [26]. Of the five hydrophobic ILs investigated, only [BMPyr^+^][Ntf_2_^-^] did not show considerable DNA uptake (which would result in a quantitative loss) or qPCR inhibition by inevitable remnants of this IL in the aqueous phase. Therefore it can be considered the ideal candidate to investigate the possible application of ILs as DNA extraction/lysis liquids.

After the establishment of a device and protocol feasible for efficient use at elevated temperatures (detailed information is available in the electronic supplementary material), the efficiency of bacterial cell lysis with changing incubation times and temperatures was investigated. To assess the general applicability of the new method, three different bacterial species (Gram-negative *E. coli* and *Salmonella* Typhimurium, and Gram-positive *L. monocytogenes*) were used, and the DNA lysis efficiency by qPCR was quantitatively compared with that of the commercially available NucleoSpin® tissue kit. Control samples prepared with the commercial kit were treated according to the manufacturer’s recommendations to achieve approximately 90% cell lysis efficiency, with a total protocol time of 3 h for *E. coli* and *Salmonella* Typhimurium and 20 h for *L. monocytogenes*.

**Fig. 3:**
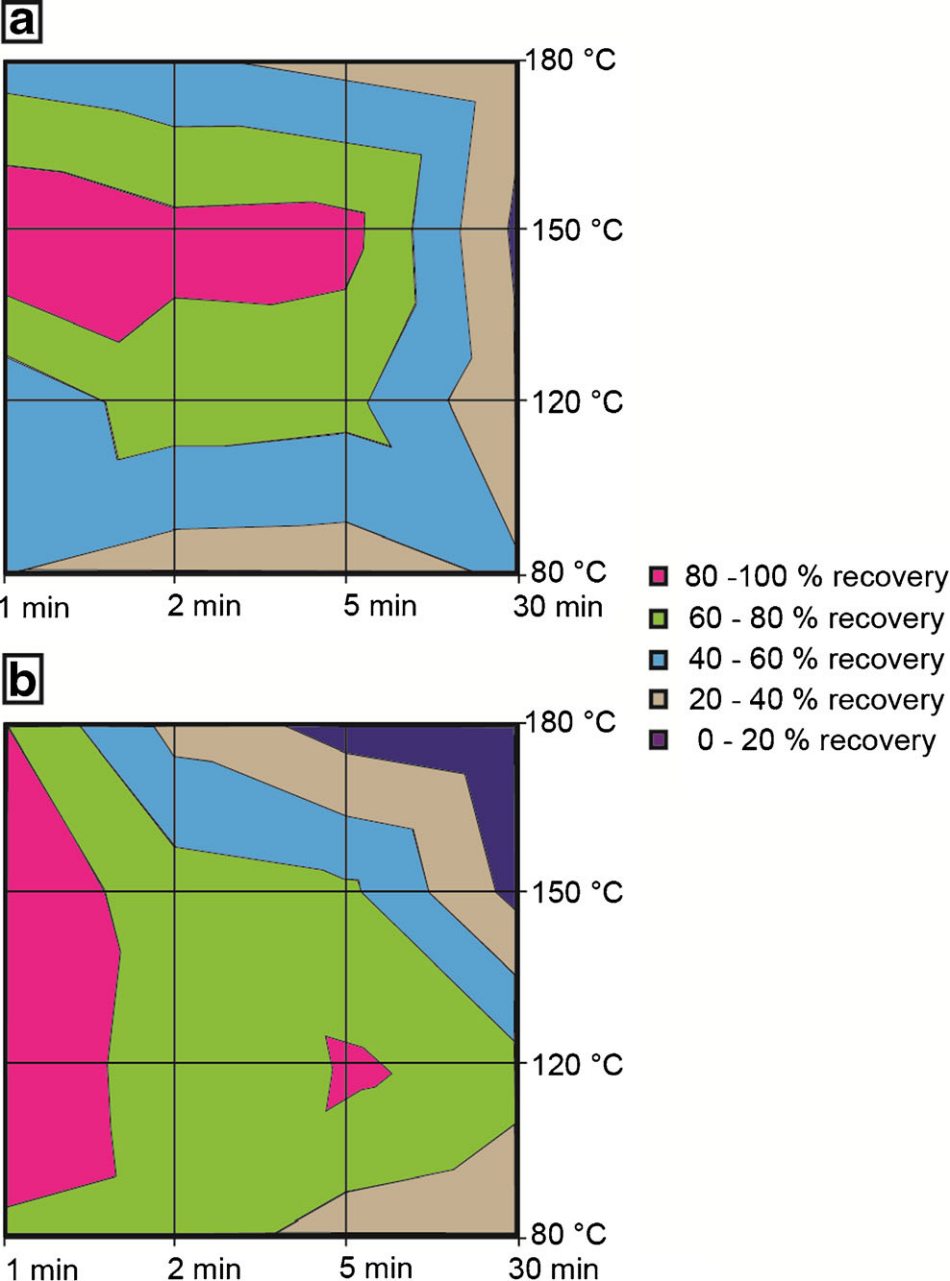
Time–temperature diagrams of the cell lysis protocol with [BMPyr^+^][Ntf_2_^-^]. For *Escherichia coli* (*A*) the highest value was measured at 1 min and 150 °C ,and for *Salmonella* Typhimurium (*B*) it was measured at 1 min and 120/150 °C. All data were calculated in proportion to the highest measured value. All samples were prepared in duplicate and a mean was calculated

The results presented in Fig. 3 show that the newly developed hydrophobic-IL-based method is able to achieve the same quantitative cell lysis values for both *E. coli* and *Salmonella* Typhimurium in 1 min as the commercial kit achieves in 3 h, whereas quantitative DNA extraction was not possible from *L. monocytogenes* (data not shown). For both *E. coli* and *Salmonella* Typhimurium, the optimal temperature for efficient cell lysis was between 120 and 150 °C. Although lower temperatures resulted in lower quantitative cell lysis efficiency, especially for *E .coli*, the highest tested temperature (180 °C) also resulted in lower values, most likely due to DNA thermal degradation. The incubation time, surprisingly, did not play a major role in terms of extraction efficiency. Indeed, the only effect observed was a loss of DNA extraction efficiency at elevated temperatures, again probably due to thermal DNA degradation.

To evaluate the purity grade of the extracted nucleic acids with regard to inhibitory effects on subsequent methods (e.g., qPCR, cloning, restriction analysis), dilution series were compared with conventionally extracted nucleic acids (NucleoSpin® tissue kit) in qPCR. As the qPCR system is one of the most sensitive molecular methods, it was assumed that other methods should not be inhibited. As quality parameters, efficiency and *R* ^2^ of the standard curve were compared, and similar values were obtained for both bacteria by the newly developed method (see Table 1).

**Table 1:** Mean cell lysis efficiency and *R* ^2^ were calculated from six independent measurements and a series of three to five dilutions was performed

	Method	Cell lysis efficiency		*R* ^2^
		Mean	RSD (%)	Mean^b^
*Salmonella* Typhimurium	Conventional^a^	96.53	1.88	0.994
	IL extraction	98.63	4.84	0.997
*Escherichia coli*	Conventional^a^	94.26	4.12	0.996
	IL extraction	88.08	4.12	0.997

*IL* ionic liquid, *RSD* relative standard deviation

^a^NucleoSpin® tissue kit

^b^Standard deviation 0.005 or less

**Fig. 4:**
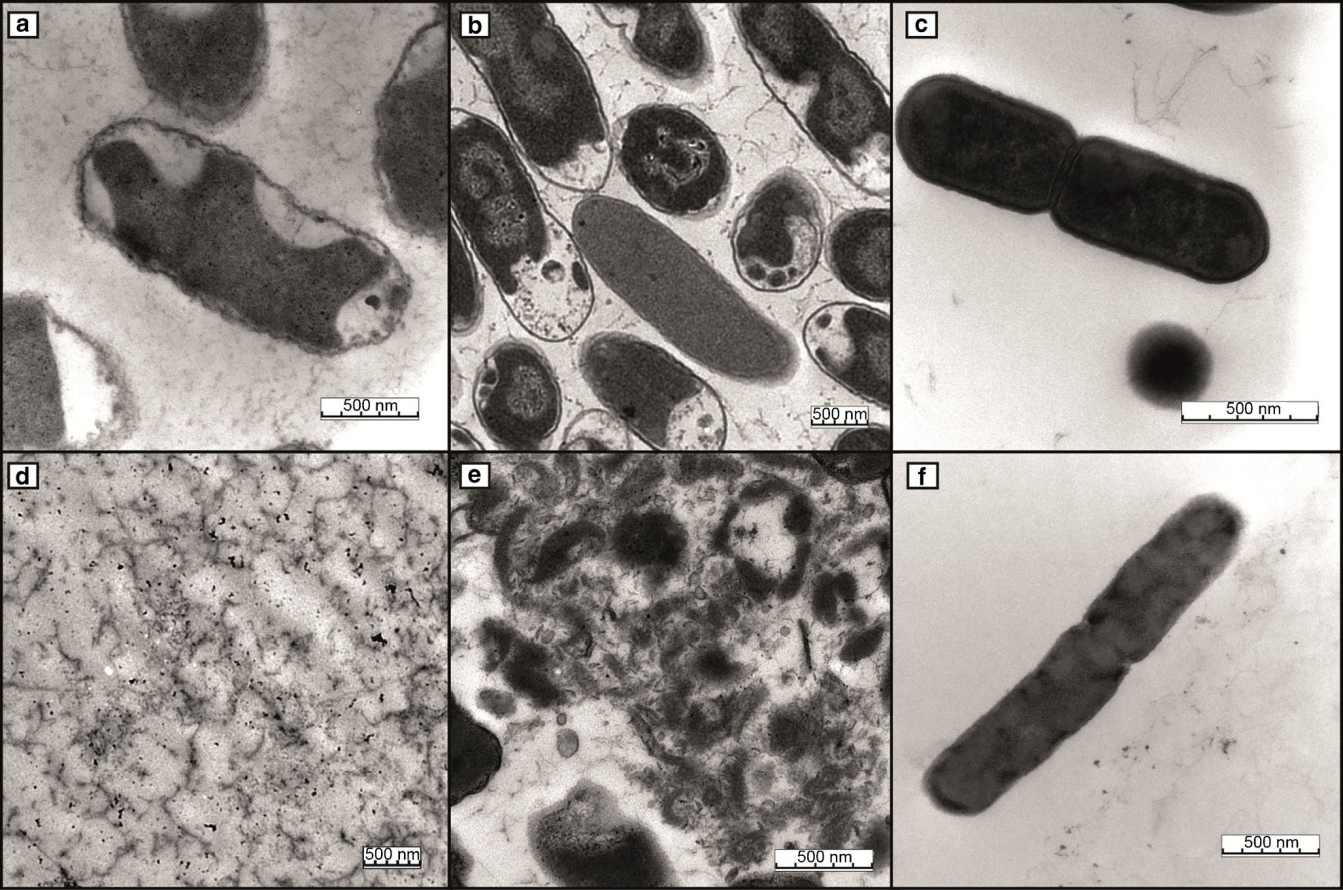
Transmission electron microscopy micrographs after the different extraction protocols with [BMPyr^+^][Ntf_2_^-^]. Micrographs of *Salmonella* Typhimurium (**a**), *E. coli* (**b**), and *Listeria monocytogenes* (**c**) controls. For the final extraction protocol, 1 min and 150 °C were selected for all three species (**d** *Salmonella* Typhimurium, **e** *E. coli*, and **f** *L. monocytogenes*). *Bars* 500 nm

In contrast to the promising results obtained by the new method for *E. coli* and *Salmonella* Typhimurium, only approximately 5% cell lysis efficiency in comparison with the commercial kit was obtained for *L. monocytogenes*. The most probable explanation for these significantly different results reflects the differences between the cell envelopes of Gram-negative and Gram-positive bacteria. Whereas the cell envelope of Gram-negative bacteria comprises two cellular lipid bilayer membranes and only a small cell wall comprising juxtaposed peptidoglycan, the cell envelope of Gram-positive bacteria is characterized by a single membrane and a much thicker outer cell wall. It is generally recognized that the thick cell wall of Gram-positive bacteria protects cellular integrity against physical and chemical stresses more effectively than the cell envelope of Gram-negative bacteria. This is also acknowledged for the NucleoSpin® tissue kit, the instructions for which recommend an additional overnight enzymatic digestion step in its support protocol for Gram-positive bacteria. To verify the influence on the cell membrane as well as the cell wall, transmission electron microscopy micrographs of the three bacterial species before and after the incubation step with [BMPyr^+^][Ntf_2_^-^] were obtained (Fig. 4). It can be observed that *Salmonella* Typhimurium and *E. coli* are completely disrupted by [BMPyr^+^][Ntf_2_^-^], whereas the cellular integrity of *L. monocytogenes* was less affected.

## Conclusion

In summary, a novel method based on hydrophobic ILs was systematically developed for bacterial cell lysis and quantitative DNA extraction for downstream molecular-biological analysis. The use of hydrophobic ILs permitted the use of elevated temperatures between 120 and 150 °C that are required for complete disintegration of Gram-negative bacteria cells. However, under these conditions, a Gram-positive species was still protected by a thick cell wall. Although no purification of genomic DNA from cellular constituents, such as carbohydrates, lipids, or salts, occurred, DNA extracted with the new protocol was of such high quality that subsequent molecular-biological methods (e.g., qPCR) could be readily used without further purification steps. This study demonstrates that hydrophobic-IL-based DNA extraction resulted in quantitative DNA extraction efficiencies for Gram-negative bacteria similar to those obtained with a commercial kit, whereas the number of handling steps, and especially the time required, was dramatically reduced. In combination with recently developed DNA concentration and purification techniques, such as magnetic IL extraction or aqueous biphasic systems, the new method has the potential to shorten significantly the time required for clinical and food diagnostics as well as high-throughput applications.

## Electronic supplementary material

Below is the link to the electronic supplementary material.

ESM 1 (PDF 227 kb)
