# Supplementary material for: Hydrophobic ionic liquids for quantitative bacterial cell lysis with subsequent DNA quantification
Source: Anal Bioanal Chem. 2016 Dec 21;409(6):1503–11. doi: 10.1007/s00216-016-0112-x (PMC5333376; doi:10.1007/s00216-016-0112-x)
Supplement: Supplementary file 1 — (PDF 227 kb) [file 216_2016_112_MOESM1_ESM.pdf]

Analytical and Bioanalytical Chemistry

Electronic Supplementary Material

Hydrophobic ionic liquids for quantitative bacterial cell lysis with subsequent DNA quantification

Fuchs-Telka Sabine, Fister Susanne, Mester Patrick, Wagner Martin, Rossmanith Peter

Quantitative PCR setup

Salmonella assay

The fimA-qPCR assay is targeting the major fimbrial subunit encoding gene *fimA* of Salmonella and amplifying a 84-bp fragment. The assay consists of the forward primer (fimAF1: 5´-CCTTTCTCCATCGTCCTGAA-3´), the reverse primer (fimAR1: TGGTGTTATCTGCCTGA-3´) and the FAM-labelled TaqMan® probe (fimAS1: 5´-FAM-TGCGATCCGAAAGTGGCGG-BHQ1-3´) (Eurofins MWG Operon, Ebersberg, Germany). All qPCRs were performed with a volume of 25 µl/PCR reaction containing a final concentration of 1× PCR buffer, 3.5 mM $MgCl_2$, 300 nM of each primer and the probe, 200 µM of dNTPs, 1.5 U Platinum® Taq DNA polymerase (Invitrogen, Vienna, Austria) and 5 µl of DNA template. A qPCR temperature profile with an initial denaturation step at 94 °C for 2 min and an amplification protocol with 45 cycles (94 °C for 30 s and 60 °C for 1 min) was used.

Listeria monocytogenes assay

The prfA-qPCR assay is targeting a 274 bp fragment of the *prfA* gene of *Listeria monocytogenes*. The assay consists of the forward primer (LIP1: 5´-GATACAGAAACATCGGTTGGC-3´), the reverse primer (LIP2: 5´-GTGTAATCTTGATGCCATCAGG-3´) and the FAM-labelled TaqMan® probe (LIP probe 2: (5´-FAM-CAGGATTAAAAGTTGACCGCA-MGB-3´) (Eurofins MWG Operon, Ebersberg, Germany).

All qPCRs were performed with a volume of 25 µl/PCR reaction containing 1x PCR buffer, 3.5 mM MgCl2, 500 nM of each primer, 250 nM of each probe, 200 µM (of dNTPs, 1.5 U of Platinum® *Taq* DNA polymerase (Invitrogen, Vienna, Austria) and 5 µl DNA template. Amplification following initial denaturation at 94 °C for 2 min was performed in 45 cycles, at 94 ◦C for 15 s, and 64 ◦C for 1 min.

Escherichia coli assay

The sfmD-qPCR assay is targeting a 106 bp fragment of *sfmD* gene, encoding a putative outer membrane export usher protein, of *E.coli*. The assay consists of the forward primer (ERT2F: 5´- ACTGGAATACTTCGGATTCAGATACGT -3´), the reverse primer (ERT2R: 5´-ATCCCTACAGATTCATTCCACGAAA-3´) and the FAM-labelled TaqMan® probe (5´-FAM-CAGCAGCTGGGTTGGCATCAGTTATTCG-BHQ1-3´) (Eurofins MWG Operon, Ebersberg, Germany).

All qPCRs were performed with a volume of 25 µl/PCR reaction containing 1x PCR buffer, 3.5 mM MgCl2, 300 nM of each primer, 200 nM of each probe, 200 µM (of dNTPs, 1 U of Platinum® *Taq* DNA polymerase (Invitrogen, Vienna, Austria) and 5 µl DNA template. Amplification following initialdenaturation at 94 °C for 2 min was performed in 45 cycles, at 94 °C for 15 s, and 60 °C for 1 min.

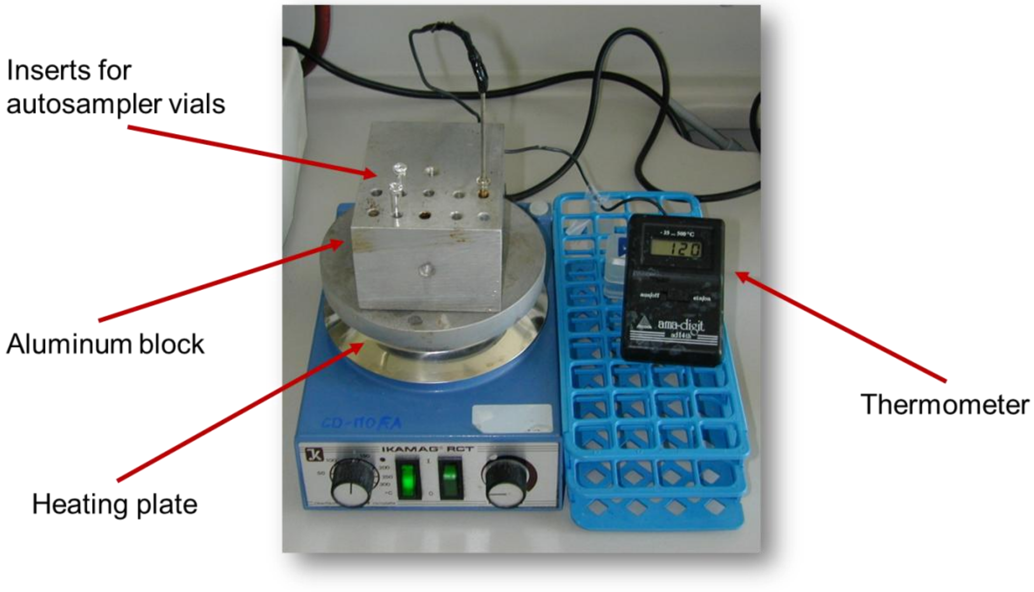

Fig S1 Aluminium block for DNA isolation experiments

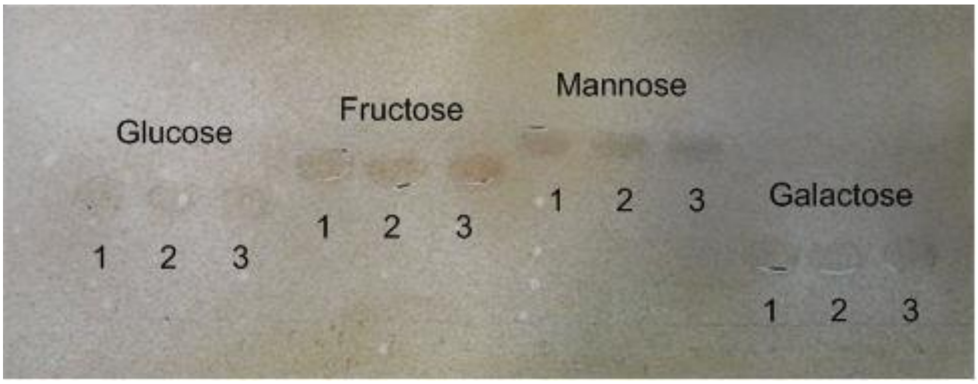

Fig. S2 Monosaccarides (glucose, fructose, mannose and galactose) applied directly (1), after extraction with $[BMPyr^{+}][Ntf_2^{-}]$, (2) or after extraction with $[BMPyr^{+}][Ntf_2^{-}]$ and additional centrifugation (5 min, 6,000 × g) (3)

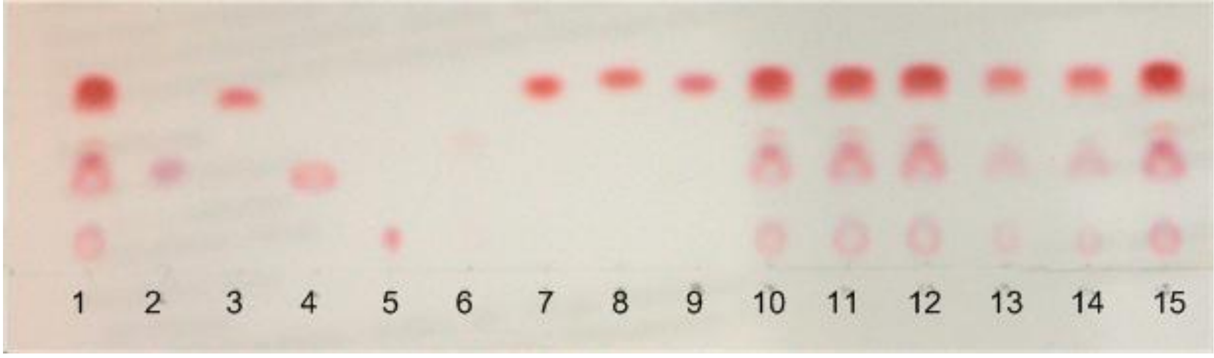

Fig. S3 Amino acids L-alanine (2), L-tyrosine (3), L-glutamine (4), L-histidine (5), L-cysteine (6), L-leucine (7), L-tryptophan (8), L-phenylalanine (9) applied directly, as a mixture containing all amino acids (1, 10 and 15), after extraction with $[BMPyr^{+}][Ntf_2^{-}]$ (11), with additional centrifugation (5 min, 6,000 × g) (12) or after extraction with $[BMPyr^{+}][Ntf_2^{-}]$ with additional incubation for 2 min at 140°C (13) and additional centrifugation (5 min, 6,000 × g) (14)
